# Hookah use patterns, social influence and associated other substance use among a sample of New York City public university students

**DOI:** 10.1186/s13011-020-00283-5

**Published:** 2020-08-28

**Authors:** Omar El Shahawy, Su Hyun Park, Erin S. Rogers, Jenni A. Shearston, Azure B. Thompson, Spring C. Cooper, Nicholas Freudenberg, Samuel A. Ball, David Abrams, Donna Shelley, Scott E. Sherman

**Affiliations:** 1 Department of Population Health, New York University School of Medicine, New York, NY USA; 2 NYU/Abu Dhabi Public Health Research Center, Abu Dhabi, United Arab Emirates; 3 School of Global Public Health, New York University, New York, USA; 4 Saw Swee Hock School of Public Health, National University of Singapore and National University Health System, Singapore, Singapore; 5 VA New York Harbor Healthcare System, New York, USA; 6 Department of Environmental Health Sciences, Mailman School of Public Health, Columbia University, New York, USA; 7 Department of Community Health Sciences, SUNY Downstate School of Public Health, Brooklyn, NY USA; 8 City University of New York Graduate School of Public Health and Health Policy, New York, USA; 9 Yale School of Medicine, Department of Psychiatry, New Haven, CT USA

**Keywords:** Hookah, Substance use, Young adults

## Abstract

**Background:**

Most hookah use studies have not included racial and ethnic minorities which limits our understanding of its use among these growing populations. This study aimed to investigate the individual characteristics of hookah use patterns and associated risk behaviors among an ethnically diverse sample of college students.

**Methods:**

A cross-sectional survey of 2460 students (aged 18–25) was conducted in 2015, and data was analyzed in 2017. Descriptive statistics were used to present the sociodemographic characteristics, hookah use-related behavior, and binge drinking and marijuana use according to the current hookah use group, including never, exclusive, dual/poly hookah use. Multivariate logistic regression was conducted to examine how hookah related behavior and other risk behaviors varied by sociodemographics and hookah use patterns.

**Results:**

Among current hookah users (*n* = 312), 70% were exclusive hookah users and 30% were dual/poly hookah users. There were no statistically significant differences in sociodemographic characteristics except for race/ethnicity (*p* < 0.05). Almost half (44%) of the exclusive hookah users reported having at least five friends who also used hookah, compared to 30% in the dual/poly use group. Exclusive users were less likely to report past year binge drinking (17%) and past year marijuana use (25%) compared to those in the dual/poly use group (44 and 48% respectively); *p* < 0.001.

**Conclusions:**

The socialization aspects of hookah smoking seem to be associated with its use patterns. Our study calls for multicomponent interventions designed to target poly tobacco use as well as other substance use that appears to be relatively common among hookah users.

## Introduction

Hookah (also called waterpipe, shisha or narghile) is one of the most commonly used combustible tobacco products by young adults in the United States [1, 2]. Hookah smokers often perceive it as safer than cigarettes [3–5], but a growing literature points to deleterious health effects, including toxicant exposure, [6] nicotine addiction and cardiorespiratory consequences [7, 8]. Existing evidence on the health effects of hookah shows it more than doubles the risk of lung cancer and respiratory illness, and case cardiovascular diseases [9, 10]. Moreover, multiple tobacco and nicotine product use (polyuse) with hookah is becoming more common [11].

Emerging adulthood (age 18–25 years) is a critical period for risk taking behavior, [13–16] and use of tobacco and other nicotine containing products has been associated with increased risk of polysubstance use, particularly alcohol and marijuana [12–15]. Among college students in the United States, the high rates of ever using hookah (42–64%) [16–18] meet or exceed the lifetime prevalence of (42%) cigarette use [17]. Initiation of hookah increases during the transition from high school to college, [19, 20] suggesting that the first few months of college is a particularly risky time for initiation [21]. Similar to drinking, the risk of rapid transition to hookah use in college may be exacerbated by the trend of social influence, as hookah is most commonly used in groups in social settings [22, 23]. Social Learning Theory and The Theory of Reasoned action emphasize the impact of socialization through peer influence and approval on substance use risk among young adults [24].

Previous studies have identified risk factors for hookah use similar to those for cigarettes (e.g., identifying as male) [25] and associated risk behaviors such as marijuana and binge drinking [19, 25]. However, most studies have not included racial and ethnic minorities limiting our understanding of use among these growing populations, this is essential in understanding patterns in risk behaviors, as the US and its college populations become increasingly diverse [26]. Nationally, less than one third (29.3%) of young adult past-30 day hookah users were exclusive hookah users [11]. Thus, it is important to understand the risk of hookah use as a function of its use pattern (i.e. exclusive vs. poly hookah and other tobacco use).

To help fill this gap, we conducted a study with a racially/ethnically diverse sample of urban college students to describe the patterns of hookah use (never, exclusive, and dual/poly use), assessed the association between hookah use patterns and perceived social acceptance and peer influence, and explored the associations between hookah use patterns and other substance use risk behavior including binge drinking and marijuana use. Our main hypothesis was that hookah-specific social influence factors assessed by perceived social disapproval of hookah smoking and number of friends who smoke hookah are associated with patterns of hookah smoking.

## Methods

### Participants

A demographically representative sample of degree-seeking students from 25 campuses of one of the United States’ largest and most diverse urban public university systems was invited to complete a health survey in 2015. It was a cross-sectional mixed-mode online and telephone survey of undergraduate and graduate students (aged 18–30). The Institutional Review Boards of New York University and City University of New York approved this study.

To ensure respondents reflected the overall student population, a stratified probability sample (*n* = 162,296) was identified based on the following criteria: type of college (4-year, technical or community college); graduate or undergraduate; race; gender; full or part-time status; number of credits completed; Grade Point Average (GPA); financial aid status; age/date of birth (under 21 or 21 to 30); and year enrolled. These criteria were used to generate post-stratification statistical weights. These weights were adjusted so that the distribution of certain weighted sample dimensions conformed to the corresponding distributions in the student population. Of the 9,990 students invited for the survey, 2987 responded (response rate 29.9%). The final strata used were Gender, Race, Class Standing, College Type and GPA Quartile. The generated strata based on the probability sampling criteria including the probability for non –response were combined to an overall weight for each participant using the Raking method. For the current analysis, we only included the subset of young adults (18–25 years, *n* = 2460) as our study population.

### Procedure

Recruitment and survey administration was conducted by the Baruch Center for Survey Research (BCSR). Potential respondents were emailed an invitation to participate in a campus wide Tobacco Use and Health Behavior Survey using a secure link. Students received up to six email reminders over the course of the data collection period. Those who did not respond after the fourth reminder were contacted (up to five attempts) by telephone by BCSR to complete the survey. The survey took on average 20 minutes to complete online and 30 minutes by telephone. Respondents received a $20 Amazon gift card for participation. The survey tool is available in Additional file 1.

### Measures

Students were asked to write their age in years and indicate their gender (male or female). We created age groups of 18–19, 20–21, 22–23, and 24–25 years. Race and ethnicity were recoded into six categories: non-Hispanic White, non-Hispanic-Black, Hispanic/Latino, Asian/Native Indian/Pacific Islander, Caribbean/West Indian, and Others. For current employment status, responses were ‘yes’ or ‘no’. Finally, we recoded household income level into three categories of: ≤ $20,000, $20,000–49,999, and ≥ $50,000.

#### Hookah smoking

Students were identified as *current hookah users* based on two measures: 1) “Which of the following products have you used in the past 30 days?” with the options of ‘Cigarettes’, ‘Cigars’, ‘Hookah’, ‘E-cigarettes’, ‘Little cigars/cigarillos’, ‘Other products not listed, please specify’ and ‘Have not used any tobacco or nicotine products in the past 30 days/none of these’ and 2) “Do you now smoke a hookah every day, some days, or not at all?” with options of ‘Every day’, ‘Some days’, ‘Not at all’ and ‘Don’t know/Not Sure’. The participants who answered either ‘Hookah’ for the first question or ‘Every day’ or ‘Some days’ for the second question were included as current hookah users. *Current hookah users* were further classified into two groups: exclusive users (those who did not report current use of other tobacco products) and dual/poly users (those who reported also using one or more other tobacco or nicotine product). Hookah never users were those who did not check “hookah” in response to the ever-use question, “Which, if any, of the following tobacco or nicotine products have you ever used or tried, even one puff?” . To contrast the risk associated with current patterns of hookah use to the least risk of never hookah use, we excluded from the analyses ever hookah users who do not currently use hookah (i.e. former hookah users) and current users of other tobacco or nicotine who do not smoke hookah, which was collectively 28 of the sample.

#### Hookah-related social influence

We assessed hookah specific social influence with two variables. *Perceived social acceptability* was measured by the question: “People who are important to you believe that you should not smoke hookah” with response categories: Strongly agree, Agree, Neutral, Disagree, Strongly disagree. We recoded the variable into yes (strongly agree, agree) and no (neutral, agree and strongly disagree). *Peer influence* was measured by the number of friends who had used a hookah: “How many of your five closest friends use hookah?” The responses were combined to produce three categorical variables of 0–2, 3–4, and 5.

#### Binge drinking and marijuana use

Binge drinking was measured among those who reported past 12 months alcohol drinking by asking the participants, “In the past 12 months, how often did you have six or more drinks on one occasion?” The choices were categorized as a binary variable: 1) Monthly, Weekly or Daily, or almost daily; and 2) Never or Less than monthly. Marijuana use was measured by asking participants, “In the past 12 months, how often have you used marijuana”. We created a binary variable to analyze marijuana use: No (Not at all) vs. Yes (Some days or Every day).

### Statistical analyses

In 2017, data were analyzed using the -svy- module in Stata 14.0 (StataCorp, College Station, TX, USA) using the default Taylor series linearization to calculate weighted percentage, with 95% CIs to account for our complex study design. Descriptive statistics for the sample were used to present the sociodemographic characteristics, hookah use-related behavior, and binge drinking and marijuana use according to the current hookah use group, including never, exclusive, dual/poly hookah use. Multivariate logistic regression models were used to investigate correlates associated with 1) exclusive and dual/poly hookah use compared to never hookah use; 2) dual/poly current use compared to exclusive hookah use. We further examined the association between exclusive and dual/poly hookah use and the following two outcomes: binge drinking and marijuana use. Model covariates included age, gender, race, current employment status, and household income, and odds ratios (ORs) and 95% confidence intervals (CIs) were estimated. Missing observations constituted less than 2% of observations (*n* = 43), thus no missing data imputation methods were used.

## Results

Out of the total analytical sample, 13% reported current hookah use. The presented analyses focused on contrasting current hookah users to never hookah users (58%), Table 1 presents the prevalence of hookah use (current exclusive, dual/poly, never) by socio-demographic characteristics. We excluded from our analyses the remaining 28% who were either current users of other tobacco or nicotine containing products or ever users of hookah with or without current use of other tobacco or nicotine containing products. Among current hookah users (*n* = 312), 70% reported exclusive hookah use, 30% reported dual/poly hookah and other tobacco product use. There were no statistically significant differences in sociodemographic characteristics by patterns of hookah use except for race/ethnicity (*p* < 0.05).

**Table 1:** Socio-demographic characteristics of the sample by hookah use patterns (never, exclusive and Dual/Poly hookah users) (*N* = 2460)

	Total (N = 2460)	Never Users (*n* = 1447)	Current users (*n* = 312)		
			Exclusive use (*n* = 219)	Dual/Poly use (*n* = 93)	*P*-value^a^
**Age**					0.234
18–19	444 (17.4)	294 (65.8)	41 (10.0)	19 (4.5)	
20–21	957 (37.1)	575 (60.0)	74 (8.0)	37 (3.6)	
22–23	663 (27.8)	371 (55.1)	75 (12.2)	22 (3.1)	
24–25	396 (17.7)	207 (51.5)	29 (7.5)	15 (3.9)	
**Gender**					0.551
Male	962 (42.9)	583 (60.3)	75 (8.5)	36 (3.5)	
Female	1477 (56.4)	852 (56.5)	143 (10.3)	53 (3.6)	
**Race/ethnicity**					0.005
NH-White	432 (18.7)	216 (49.9)	33 (8.2)	25 (5.9)	
NH-Black	297 (12.8)	207 (68.8)	19 (6.5)	1 (0.5)	
Hispanic/Latino	429 (18.6)	223 (51.9)	57 (14.0)	23 (5.2)	
Asian/ NI/PI	624 (22.2)	424 (66.9)	29 (4.9)	18 (2.8)	
Caribbean/West Indian	355 (14.7)	193 (53.7)	52 (15.0)	9 (2.4)	
Other	308 (12.4)	178 (59.1)	26 (8.8)	17 (4.6)	
**Household income**					0.136
< $20,000	700 (28.9)	455 (64.3)	65 (10.0)	19 (2.6)	
$20,000 - $49,999	747 (30.0)	448 (57.9)	72 (10.8)	33 (4.4)	
> $50,000	728 (28.6)	349 (47.7)	66 (9.2)	35 (4.7)	
**Currently employed**					0.189
No	1066 (42.7)	716 (66.6)	71 (6.8)	37 (3.3)	
Yes	1354 (55.7)	711 (51.8)	145 (11.6)	55 (4.0)	

NH, Non-Hispanic;

Values are n(%)

Row percentages do not add up to 100% as the category of ever hookah users and current other tobacco users (*n* = 658) and missing responses (n = 43) are not included in the table

^a^Chi-square test between exclusive hookah users vs. dual/poly hookah users

About half of the exclusive hookah users (47%) reported they perceived social disapproval for their hookah smoking, compared to 30% dual/poly users (*p* < 0.05). In addition, 44% of those reporting exclusive hookah smoking also reported having at least five friends who also used hookah, compared to 30% in the dual/poly use group (Table 2). Exclusive hookah users were less likely to report past-year binge drinking (17%) compared dual/poly users (44%); *p* < 0.001. Similarly, exclusive hookah users were less likely to report past year marijuana use (25%) compared to those in the poly use group (48%); *p* < 0.001.

**Table 2:** Hookah use social context and alcohol and marijuana use by hookah use patterns (never, exclusive and dual/poly hookah users) (*N* = 2460)

	Total (*N* = 2460)	Never Users (*n* = 1447)	Current users (n = 312)		
			Exclusive use (*n* = 219)	Dual/Poly use (*n* = 93)	*P*-Value^a^
**Perceived Disapproval of Hookah Smoking**^**b**^					0.008
Yes	1576 (63.5)	1076 (67.3)	102 (7.0)	28 (1.7)	
No	865 (35.7)	363 (42.2)	116 (13.9)	65 (7.2)	
**Close friends use of hookah**^**c**^					0.042
0–2	1637 (65.9)	1086 (65.7)	70 (4.6)	35 (2.2)	
3–4	317 (13.0)	146 (45.8)	48 (15.5)	28 (8.7)	
5	322 (13.6)	92 (28.3)	96 (31.3)	28 (8.0)	
**Past Year Binge Drinking**^**d**^					0.0001
No	1352 (55.2)	707 (51.8)	151 (11.9)	47 (3.5)	
Yes	279 (12.0)	88 (33.2)	32 (12.2)	38 (12.0)	
**Past Year Marijuana use**					0.0001
No	2005 (81.0)	1322 (65.2)	163 (8.8)	46 (2.3)	
Yes	413 (17.4)	101 (25.0)	55 (13.4)	44 (10.1)	

Row percentages do not add up to 100% as the category of ever hookah users and current other tobacco users (n = 658) and missing responses (n = 43) are not included in the table

^a^Chi-square test between exclusive hookah users vs. dual/poly hookah users

^b^Disapproval by people who are important to the respondent “People who are important to you believe that you should not smoke hookah”

^c^Close friends use of hookah was defined as the friends that you spend time with on a regular basis

^d^Binge drinking is reported among students with reported past 12-month history of alcohol drinking

Socio-demographic and social influence factors associated with exclusive and dual/poly hookah use in multivariate logistic regression analyses are shown in Table 3. Students who reported being currently employed were more likely to be current exclusive hookah users (aOR = 1.85; 95% CI = 1.27–2.69) compared to never hookah users. Students who reported being Caribbean/West Indian were less likely to be dual/poly hookah users than either never hookah users (aOR = 0.35; 95% CI = 0.14–0.88) or exclusive hookah users (aOR = 0.23; 95% CI = 0.08, 0.67) compared to non-Hispanic Whites Table 3.

**Table 3:** Multivariate association of the sociodemographic and social influence factors by hookah use patterns (never, exclusive and Dual/Poly hookah users) (*n* = 1759)^a^

	Exclusive hookah use vs. Never hookah use (ref) (*N* = 1538)	Dual/Poly hookah use vs. Never hookah use (ref) (*N* = 1415)	Dual/Poly hookah use vs. Exclusive hookah use (ref) (*N* = 305)
	AOR (95% CI)	AOR (95% CI)	AOR (95% CI)
**Age**			
18–19	1.00	1.00	1.00
20–21	0.97 (0.57, 1.63)	0.87 (0.41, 1.84)	1.08 (0.47, 2.52)
22–23	1.50 (0.90, 2.50)	0.81 (0.36, 1.82)	0.53 (0.23, 1.25)
24–25	1.04 (0.56, 1.96)	1.08 (0.40, 2.88)	1.80 (0.60, 5.42)
**Gender**			
Male	0.84 (0.57, 1.96)	0.90 (0.52, 1.57)	0.97 (0.52, 1.79)
Female	1.00	1.00	1.00
**Race/ethnicity**			
NH-White	1.00	1.00	1.00
NH-Black^b^	0.61 (0.29, 1.26)	N/A^b^	N/A^b^
Hispanic/Latino	1.56 (0.30, 1.06)	0.97 (0.43, 2.19)	0.54 (0.23, 1.25)
Asian/ NI/PI	0.56 (0.30, 1.06)	0.53 (0.25, 1.13)	1.20 (0.49, 2.95)
Caribbean/West Indian	1.27 (0.69, 2.34)	0.35 (0.14, 0.88)*	0.23 (0.08, 0.67)**
Other	0.75 (0.37, 1.51)	0.64 (0.27, 1.55)	1.04 (0.39, 2.78)
**Currently employed**			
Yes	1.85 (1.27, 2.69)**	1.34 (0.79, 2.29)	0.68 (0.37, 1.28)
No	1.00	1.00	1.00
**Household income**			
< $20,000	0.87 (0.56, 1.37)	0.60 (0.30, 1.21)	0.72 (0.33, 1.57)
$20,000 - $49,999	1.00	1.00	1.00
> $50,000	1.05 (0.67, 1.67)	1.25 (0.65, 2.40)	1.11 (0.55, 2.22)
**Perceived Social Disapproval of Hookah Smoking**^**c**^			
Yes	1.00	1.00	1.00
No	1.72 (1.18, 2.52)**	4.04 (2.27, 7.19)**	3.14 (1.64, 6.02)**
**Close Friends use of hookah**^**d**^			
0–2	1.00	1.00	1.00
3–4	3.89 (2.47, 6.12)**	5.11 (2.67, 9.80)**	1.53 (0.70, 3.33)
5 or more	12.43 (7.96, 19.39)**	6.65 (3.27, 13.54)**	0.52 (0.26, 1.05)

*AOR* adjusted odds ratio, *CI* confidence interval, *NH* non-Hispanic, *NI* native indians, *PI* pacific islander

*p < 0.05, **p < 0.01

^a^This analysis excludes exclusive tobacco users and former hookah smokers

^b^This category is not included in the analyses due to the small number (n = 1)

^c^Perceived social disapproval of hookah smoking was ascertained as a binary variable by assessing participants’ agreement to the statement “People who are important to you believe that you should not smoke hookah”

^d^Close friends was defined as the friends that you spend time with on a regular basis

For the social influence factors, students who reported having three or four close friends who use hookah were more likely to be exclusive or dual/poly users than never users (aOR = 3.89; 95% CI = 2.47–6.12, aOR = 5.11; 95% CI = 2.67–9.80, respectively), compared to those who reported having two friends or less who use hookah. Moreover, students who reported having five close or more friends compared to having two friends or less who use hookah, were more likely to be exclusive or dual/poly users than never users (aOR = 12.43; 95% CI = 7.96–19.39, aOR = 6.65; 95% CI = 3.27–13.54, respectively). Those who perceived lower disapproval from important people were more likely to be hookah users (exclusive or dual/poly) than non-users in contrast to those who perceived higher disapproval (aOR = 1.72, 95% CI = 1.18, 2.52; aOR = 4.04, 95% CI = 2.27, 7.19 respectively). Similarly, dual/poly hookah users who perceived that people important to them would not disapprove of their hookah smoking, were more likely to smoke hookah compared to exclusive hookah users in contrast to those who perceived higher disapproval (aOR = 3.14, 95% CI = 1.64, 6.02).

Associations between hookah use, binge drinking, and marijuana use are shown in Table 4. Students who reported current use of hookah, regardless of the pattern (i.e. exclusive or dual/poly use), were more likely to use marijuana in the past year, as compared to students who reported never using hookah (aORs range: 2.34–6.65). Similarly, students who reported exclusive or dual/poly use of hookah were more likely to report past year binge drinking, compared to never hookah users, as well as those reporting dual/poly hookah use compared to exclusive hookah use (aORs range: 1.76–7.30).

**Table 4:** Multivariate association between hookah use patterns and binge drinking and marijuana use among those reporting past 12 months alcohol and marijuana use

		Past Year Binge drinking		Past Year Marijuana use
	N	AOR (95% CI)	N	AOR (95% CI)
**Exclusive hookah use vs. Never hookah use (ref)**	978	1.76 (1.02, 3.04)*	1641	2.34 (1.44, 3.78)**
**Dual/poly hookah use vs. Never hookah use (ref)**	880	7.30 (3.87, 12.79)**	1513	6.65 (3.65, 12.10)**
**Dual/poly hookah use vs. Exclusive hookah use (ref)**	268	5.29 (2.52, 11.08)**	308	3.24 (1.68, 6.26)**

Adjusted for age, gender, race, income, current employment status, close friends use of hookah, and perceived Social Disapproval of Hookah Smoking

*p < 0.05, **p < 0.01

## Discussion

Among a racially/ethnically diverse sample of young adults in one of the largest public urban university systems in the US, in contrast to prior studies, use of other tobacco and nicotine products along with hookah was less prevalent than exclusive hookah use among our young adult sample [27]. Patterns of hookah use were significantly associated with past year alcohol binge drinking and marijuana use, where dual/poly hookah users were more likely to report binge drinking and marijuana use more than exclusive hookah use with never hookah users as the reference group.

Historically, young adult males have had higher estimates of hookah use compared to females [28]; however, trends have started changing with recent reports indicating that male and female prevalence is comparable [20]. In contrast to the overall national US prevalence estimates, current hookah smoking was not significantly different between males and females, even when analyzed by hookah use pattern [29]. However, there is a growing appeal for hookah smoking among urban female college students, including use of multiple tobacco and nicotine products in addition to hookah [11]. Hookah is thought to be emerging during young adults’ transition to college, especially among females, which could be perceived a sign of independence among college age students [19]. Hookah bars are usually located around college campuses [22], and where there is potentially high density of college students, particularly around the campuses included in our study. Around 121 out of 137 (88%) of the hookah bars in New York State are present in the 5 boroughs [30] where the campuses included in our study are located. Given that females are reportedly more likely to smoke hookah in cafes/hookah bars compared to males [11], this high density of hookah bars might be a contributing risk factor for the higher hookah smoking prevalence among young adult females in our study compared to the national US estimates.

Race/ethnicity and social influences were independently associated with patterns of current hookah use. One significant difference by race/ethnicity was that Caribbean/West Indian origin students were less likely to be dual/poly users of hookah. We did not find higher hookah use rates among young adult White and Hispanic populations reported in other studies [19, 31]. This may be due to the unique pattern of smoking behavior within New York City, which limits hookah use disparities among college students. Future studies should assess in depth the racial and ethnic differences within patterns of hookah use while accounting for a more comprehensive set of hookah use predictors.

Social influence factors were significantly associated with most of the patterns of hookah use (exclusive and dual/poly). These findings are consistent with the prior literature of substance use, as well as hookah use, which reflects a strong context for hookah use as a social activity [22, 23]. However, this finding can be interpreted in two ways; social influence could be impacting hookah use in general and whether an individual uses hookah exclusively or in combination with other products; a novel finding in the hookah literature. Moreover, having a greater number of friends who use hookah was more strongly associated with exclusive hookah smoking in contrast to dual/poly hookah smoking. On the other hand, indviduals who elect to use hookah may be prone to seek friends with similar behaviors (i.e. smoking hookah); especially since smoking hookah usually occurs in groups. Thus, future interventions could benefit from addressing groups rather than individuals; for example, by promoting and facilitating other group social activities and interactions to replace hookah-smoking gatherings.

As hypothesized and consistent with prior studies [32], we found an incremental risk of past year binge drinking and marijuana use associated with current exclusive and dual/poly hookah user. Dual/poly hookah users had significantly higher odds of past year binge drinking and marijuana use, in contrast to never hookah users and exclusive hookah users. However, these associations may have been inflated due to limiting the comparisons between the least risky group of never users of hookah to the current dual/poly hookah users. Consistent with Problem Behavior Theory, risk behaviors assessed in our study clustered together [33]. These findings suggest that current dual/poly hookah use relates to higher risk of binge drinking and marijuana use and reflects an emerging constellation of risky behaviors that might benefit from a brief intervention. Second, these results imply that many hookah users do not only need assistance with abstaining from hookah, but may need assistance abstaining from other forms of tobacco, as well as binge drinking and marijuana. Interventions and policies designed for prevention and cessation of hookah use may have an impact on other substances such as alcohol and marijuana use. Nevertheless, this also suggests that there is a need for cessation interventions that are designed to address multiple substances [34].

Hookah use continues to spread among young adults. To help reduce tobacco initiation among young adults, there has been growing advocacy and support for raising the tobacco purchasing age to 21 years old in the US [35]. New York City was one of the first cities to implement this ban [36]; this provision does not yet apply to hookah use and access to hookah bars. Given that the average age of initiation for hookah in the US is 18 years old [11], current regulations that raised the minimum purchasing age of other tobacco products to 21 should include clear provisions on types of tobacco covered to include hookah as well (currently, the minimum age for hookah sales is still 18 years old) [37], and also to include hookah tobacco and bars and cafes where hookah is served. Enforcing such regulations might also be particularly more impactful among young adult females transitioning to college, as they are more likely to be using hookah in cafes compared to young adult males [11].

### Limitations

This is the one of the first studies we are aware of, which assesses patterns of hookah use among a racially/ethnically diverse college population including a large racial/ethnic minority group and its association with binge drinking and marijuana use. Limitations include a relatively low (30%) survey response rate. It did not account for other predictors of tobacco and substance use, such as religiosity or sensation seeking [38, 39], that might interfere with some substance use assessed in our study [40]. The analyses were based on self-reported data; recall and social desirability biases may have affected the results. We did not include former hookah users in the final analyses, this group may have had different patterns of use and risk behavior that our current analyses did not address. The survey utilizes cross-sectional data; therefore, it was not possible to assess the causality of relationships. There are potentially other important unexplored variables in this assessment of hookah use patterns. In addition, patterns of hookah or other tobacco use may be different in university systems with substantial suburban or rural populations, limiting the generalizability of these findings to other college populations.

## Conclusions

The socialization aspects of hookah smoking seem to not only impact exclusive hookah use, but also dual/poly hookah use. Future research should evaluate how other substances are being used among young adult hookah smokers. Given that there are no existing hookah use specific cessation interventions targeting young adults [41], there is a missed opportunity for targeting this diverse and vulnerable population. This reflects a need for scalable smoking cessation interventions addressing the different patterns of hookah use among young adults. Finally, our study calls for multicomponent interventions designed to target multiple forms of tobacco use, as well as other substance use that appears to be relatively common among hookah users [42].

## Supplementary information

**Additional file 1.**

## Data Availability

Data used for this study can be accessed upon request from the Principal Investigator (Dr. Scott Sherman) at scott.sherman@nyulangone.org.

## Abbreviations

GPA: Grade Point Average
BCSR: Baruch Center for Survey Research
OR: Odds Ratio
CF: Confidence Interval
AOR: Adjusted Odds Ratio
