# Supplementary material for: Hookah use patterns, social influence and associated other substance use among a sample of New York City public university students
Source: Subst Abuse Treat Prev Policy. 2020 Aug 28;15:65. doi: 10.1186/s13011-020-00283-5 (PMC7453717; doi:10.1186/s13011-020-00283-5)
Supplement: Supplementary file 1 — Additional file 1. [file 13011_2020_283_MOESM1_ESM.docx]

**Additional file 1: Initial CUNY Survey** (Short form for questions included in the publication)

CUNY-SPECIFIC COLLEGE ATTENDANCE

**First, we will ask you some questions about your college experience, specifically at CUNY.**

A1. Which CUNY campus are you enrolled at primarily? **(Select only ONE answer)**

- 1. Baruch
  2. BMCC
  3. Bronx CC
  4. Brooklyn C
  5. City C
  6. College of Staten Is.
  7. Graduate Center
  8. Graduate School of Journalism
  9. Hostos CC
  10. Hunter C
  11. John Jay C
  12. Kingsborough CC
  13. La Guardia CC
  14. Lehman C
  15. Macaulay Honors C
  16. Medgar Evers C
  17. NYC Tech
  18. Queens C
  19. Queensborough CC
  20. School of Law
  21. School of Professional Studies
  22. Stella and Charles Gutterman CC
  23. York C
  24. Other

A2. What is your current class standing?

1. First-year student
2. Second-year student
3. Third-year student
4. Fourth-year student
5. Graduate student - Master’s program
6. Graduate student - Doctoral program
7. Other, please specify:______________

A3. What is your overall cumulative GPA at CUNY?__________

DEMOGRAPHICS

**These next questions will ask you about some of your social characteristics.**

B1. How old are you? ______ YEARS

B2. What gender do you consider yourself?

- 1. Male
  2. Female
  3. Transgender
  4. Other

B3. Are you Hispanic or Latino?

1. Yes
2. No

B4. Which of the following best describes you?

1. White
2. African-American/ Black
3. Asian/ Asian American
4. Caribbean or West Indian
5. Arab or Middle-Eastern
6. African
7. American Indian or Alaska Native
8. Native Hawaiian or Pacific Islander
9. Other, please describe: ___________

______________________________

B5. Were you born in the United States?

- 1. Yes
  2. No
  3. Don’t know/Unsure

B6. Are you…

1. Single?
2. Married?
3. Dating?
4. Separated?
5. Divorced?
6. Widowed?

B7. Are you a parent or guardian of any children?

1. Yes → Go to B7a
2. No → Go to B8
3. Don’t know/Unsure → Go to B8

[If YES,] →

B7a. How many children under age 18 live with you now? _______CHILDREN

B8. What is your current home zip code: ________

GENERAL HEALTH

**Next, we would like to ask about your general health and health insurance status.**

C1. Would you say that in general your health is…

1. Excellent
2. Very good
3. Good
4. Fair
5. Poor

C2. What is your current height?

______FT _____ INCHES

C3. What is your current weight?

_______ LBS

C4. Do you consider yourself…

1. Underweight
2. Normal weight
3. Overweight
4. Obese
5. I haven’t really considered it

C5. In the past 12 months, have you tried to lose weight?

- 1. Yes
  2. No

C6. In the past 12 months, have you seen a medical doctor?

1. Yes
2. No

C7. In the past 12 months, have you seen a dentist?

1. Yes
2. No

C8. Are you currently covered by any health insurance?

- 1. Yes
  2. No

ALL TOBACCO (EVER/ CURRENT USE)

The following questions will be about using any tobacco or nicotine products during your lifetime.

D1. Which, if any, of the following tobacco or nicotine products have you ever used or tried, even one puff? **Select all that apply. IF SKIPPED, GO TO D1a**

1. Cigarettes
2. Cigars
3. Hookah/shisha/waterpipe/narghile
4. Electronic-cigarettes, vape pens or personal vaporizers containing nicotine (e.g. Blu, NJOY, or ‘mods’). These might also include e-cigars, e-pipes, e-hookahs, and hookah pens. We are NOT including devices that vaporize pot or marijuana.
5. Little cigars/cigarillos (e.g. Black & Milds)
6. Other product not listed, specify: ___________________________
7. Have never used tobacco or nicotine products/ None of these → Go to D2

[IF SELECTED A THROUGH G in D1,] →

D1a. The first time you tried tobacco or nicotine, what type did you try? **Select only one response**.

1. Cigarettes
2. Cigars
3. Hookah
4. E-cigarettes
5. Little cigars/cigarillos
6. Other product not listed, please specify: ___________________________
7. Not applicable → Go to D1b

[SELECTIONS BASED ON QB1. PRODUCTS EVER USED] →

D1b. Which of the following products have you used in the past 30 days? **Select all that apply.**

- - 1. Cigarettes
    2. Cigars
    3. Hookah
    4. E-cigarettes
    5. Little cigars/cigarillos
    6. Other product not listed, please specify: _____________________
    7. Have not used any tobacco or nicotine products in the past 30 days/None of these → Go to D2

INTENTION

**For E6-8: IF ANSWERED NEVER USED HOOKAH IN D1 OR SKIPPED D1**

**The next few questions are about smoking tobacco in a hookah. As a reminder, a hookah pipe is a type of water pipe that is sometimes also called a narghile pipe. See pictures below for examples.**


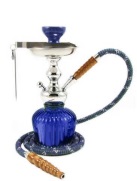

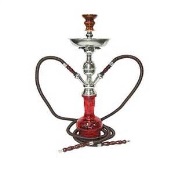


**People smoke shisha or hookah tobacco in a hookah. Some shisha contains tobacco and some does not. Shisha comes in many flavors. See pictures below for examples.**


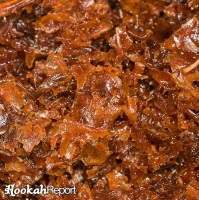

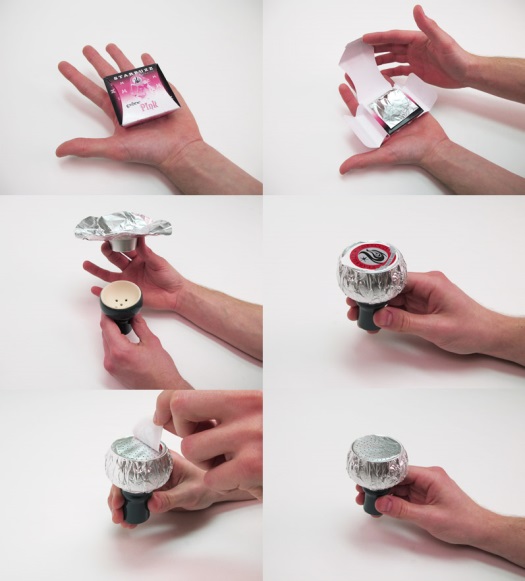

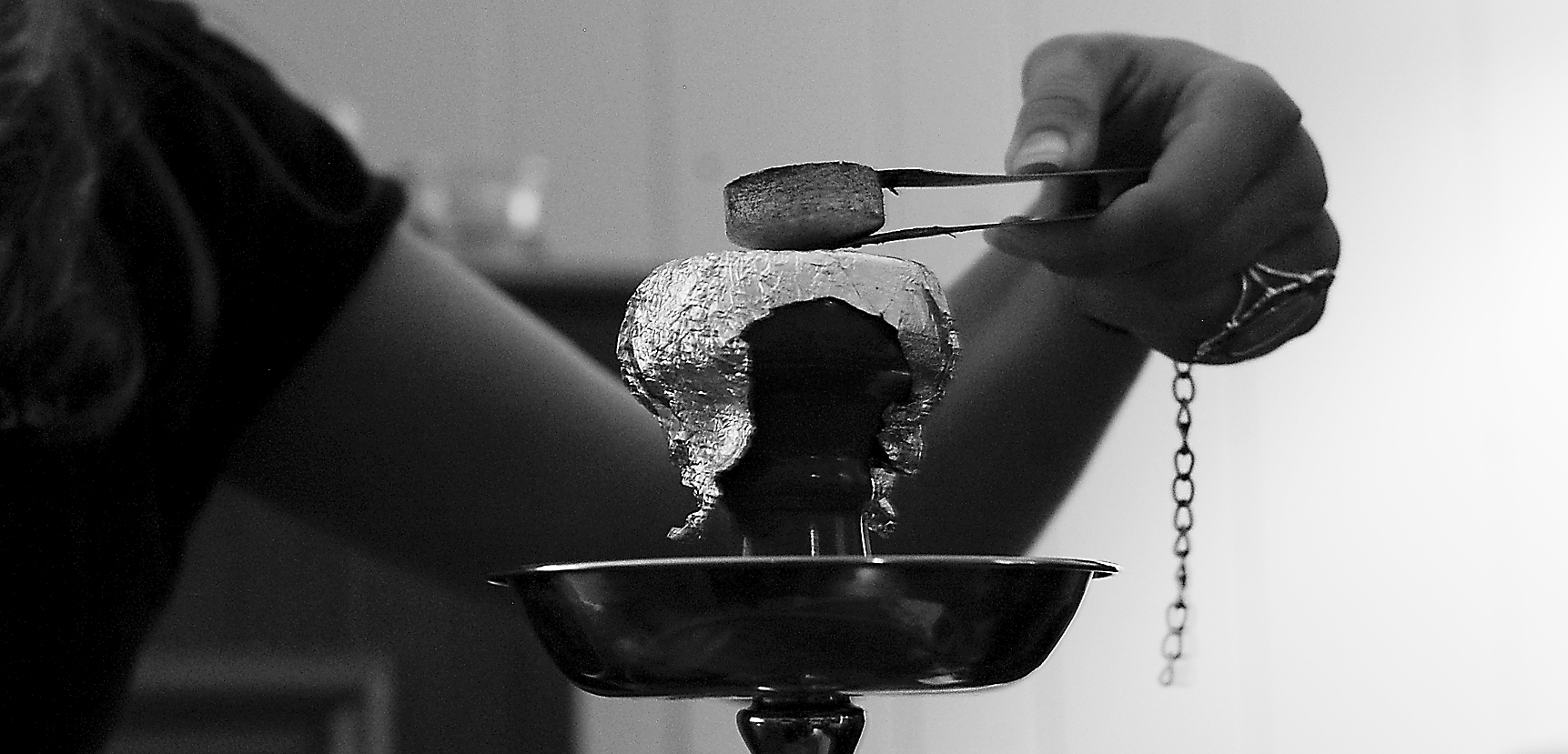


E8. If one of your friends or somebody close to you offered you hookah, would you try it? Would you say…

1. Definitely yes
2. Probably yes
3. Probably not
4. Definitely not

CIGARETTE USERS

F1. Have you smoked at least 100 cigarettes (*approximately 5 packs*) in your entire life?

1. Yes 🡪 Go to F2
2. No 🡪 Go to G1 or H1
3. Don’t Know/Not Sure 🡪 Go to F2
4. I’ve never smoked cigarettes/Not applicable 🡪 Go to G1 or H1

F2. Do you now smoke cigarettes every day, some days, or not at all?

1. Every Day 🡪 Go to F2a
2. Some Days 🡪 Go to F2b
3. Not At All 🡪Go to F3
4. Don’t Know/Not Sure 🡪Go to F3

HOOKAH USERS

**For G1-G4, IF ANSWERED EVER USED HOOKAH IN D1 OR SKIPPED D1**

G1. How many times have you smoked a hookah in your entire life?

1. 1 to 10 times
2. 11 to 20 times
3. 21 to 50 times
4. 51 to 99 times
5. At least 100 or more times
6. I’ve never smoked hookah/Not applicable 🡪 Go to H1

G2. Do you **now** smoke hookah every day, some days, or not at all?

1. Every day 🡪 Go to G2b
2. Some days 🡪 Go to G2a
3. Not at all 🡪Go to G3
4. Don’t Know/Not Sure 🡪Go to G3

[IF SOME DAYS] →

G2a. During the last 30 days, on how many days did you smoke hookah?

____ DAYS

[IF SOME DAYS or EVERY DAY] →

G2b. On the days you smoke hookah, about how many hookah sessions or pipes do you smoke each day?

_____ HOOKAH SESSIONS

G2c. Compared to one year ago, are you currently smoking hookah:

1. More now than last year
2. The same as last year
3. Less than last year
4. Did not smoke hookah one year ago

G10. Where [do / did] you usually smoke a hookah? **Select all that apply.**

1. At home
2. In a bar or cafe
3. At a friend’s house
4. Somewhere else, please specify:________________

G11. Do you usually share the same hookah with others?

- 1. Yes
  2. No

G12. Is the hookah you smoke most often flavored to taste like...

1. Menthol or mint
2. Clove or spice
3. Candy, chocolate, or other sweets
4. Fruit
5. Alcohol (such as wine or cognac)
6. Tobacco/ not flavored
7. Other, please specify:________________

ELECTRONIC-CIGARETTE USERS

H1. Do you **now** use e-cigarettes every day, some days, or not at all?

1. Every day 🡪Go to H2
2. Some days 🡪Go to H1a
3. Not at all 🡪Go to H4
4. Don’t Know/Not Sure 🡪Go to H4
5. I’ve never used e-cigarettes/Not applicable 🡪Go to I1

[IF SOME DAYS] →

H1a. During the last 30 days, on how many days did you use e-cigarettes? Please enter number of days.

______ DAYS

H2. Do you usually use an e-cigarette that is rechargeable or disposable?

1. Rechargeable e-cigarette 🡪Go to H2c
2. Disposable e-cigarettes 🡪Go to H2a

[IF DISPOSABLE] →

H2a. Think about the last 30 days… About how many separate times did you use your e-cigarette on the days you use it?

_____ TIMES PER DAY

H2b. During each of those times that you used your e-cigarette, approximately how many puffs do you take?

1. ________ PUFFS PER USE

[IF RECHARGABLE] →

H2c. Think about the last 30 days … About how many separate times did you use your e-cigarette on the days you use it?

_____ TIMES PER DAY

H2d. During each of those times that you used your e-cigarette, approximately how many puffs do you take?

________ PUFFS PER USE

PEER AND FAMILY INFLUENCES

**For K1, IF ANSWERED USED CIGARETTES/E-CIGARETTES/HOOKAH IN PAST 30 DAYS IN D1b**

K1. In the past 12 months, to what extent, if at all, [has/did] the disapproval of close friends and family of your smoking [led/lead] you to think about quitting __[cigarettes/ e-cigarettes/ hookah] _____?

1. Not at all
2. Somewhat
3. Very much

**For K2-K, FOR ALL RESPONDENTS**

K2. People who are important to you believe that you should not smoke cigarettes.

- - 1. Strongly agree
    2. Agree
    3. Neutral
    4. Disagree
    5. Strongly disagree

K3. People who are important to you believe that you should not smoke hookah.

1. Strongly agree
2. Agree
3. Neutral
4. Disagree
5. Strongly disagree

K4. People who are important to you believe that you should not use e-cigarettes.

1. Strongly agree
2. Agree
3. Neutral
4. Disagree
5. Strongly disagree

Please think of your **five closest friends** that you spend time with on a regular basis. How many of them use….

|  | **Please enter number of friends (*up to 5*)** |
| --- | --- |
|  |  |
| K5. Cigarettes? |  |
| K6. Hookah? |  |
| K7. E-Cigarettes? |  |

K8. Do any of your immediate family members (parents, brothers, sisters) smoke hookah?

- - 1. Yes
    2. No

RISK PERCEPTION

**For L1-L4, FOR ALL RESPONDENTS**

L1. How harmful do you think cigarettes are to health?

- 1. Not at all harmful
  2. Slightly harmful
  3. Somewhat harmful
  4. Very harmful
  5. Extremely harmful

**Compared to cigarettes, how harmful do you think the following products are to a person’s health?**

|  | a. A lot less harmful | b. A little less harmful | c. About the same | - 1. A little more harmful | - 1. A lot more harmful |
| --- | --- | --- | --- | --- | --- |
| L2. Hookah | □ | □ | □ | □ | □ |
| L3. E-cigarettes | □ | □ | □ | □ | □ |
| L4. Marijuana | □ | □ | □ | □ | □ |
| L5. Alcohol 🡪 Go to L6 or M1 | □ | □ | □ | □ | □ |

ALCOHOL

**For N1-N1d, FOR ALL RESPONDENTS**

N1. Within the past 12 months, have you consumed alcohol?

1. Yes
2. No 🡪Go to O1

[If YES,] →

N1a. In the last 12 months, about how often have you had a drink containing alcohol?

1. Everyday
2. 3 or more times a week
3. 1-2 times a week
4. 1-2 times a month
5. Once every couple of months or less

N1b. On a typical day when you are drinking, about how many alcoholic drinks do you have? **By a drink, we mean a glass of wine, wine cooler, can or bottle of beer, shot of liquor, or mixed drink.**

___________ DRINKS

N1c. On a typical day when you are drinking, about how many hours do you spend drinking from your first to your last drink?

_________ HOURS

N1d. In the past 12 months, how often did you have six or more drinks on one occasion?

- - - - 1. Never
        2. Less than monthly
        3. Monthly
        4. Weekly
        5. Daily or almost daily

N1e. In the last 12 months, how much money did you spend on alcohol **during a typical month**?

$________ PER MONTH

DRUG USE

**The following questions ask about use of drugs in recreational ways. Please remember that your answers to these questions are strictly confidential.**

**For O1-O4, FOR ALL RESPONDENTS**

**In the past 12 months, how often, *if ever*, have you used the following…**

|  | a. Not at all | b. Some days | c. Every day |
| --- | --- | --- | --- |
| O1. Marijuana | □ | □ | □ |
| O2. Other drugs (cocaine, heroin, ecstasy/molly, meth, etc.) | □ | □ | □ |
| O3. Prescription drugs (not prescribed for you or used in an unprescribed manner) | □ | □ | □ |

O4. Do you know someone who has experienced an opioid overdose? (This could be a friend, a family member, or yourself.)

1. Yes
2. No
3. Unsure

**For Q9-Q19, FOR ALL RESPONDENTS**

Q9. Have you ever served in the United States Armed Forces?

1. Yes
2. No

Q10. Are you currently employed?

- 1. Yes → Go to Q10a
  2. No → Go to Q11

[If YES,] →

Q10a. On average, how many hours a week did you work in the past 12 months?

- 1. 1-19 hours per week
  2. 20-34 hours per week
  3. 35 or more hours per week (full-time)

Q11. What is your main source of income?

- 1. My work
  2. Parents’/guardians’ work
  3. Spouse’s/partner’s work
  4. Student loans/fellowships
  5. Public benefits
  6. Investments
  7. Other, please specify: ____________________________

Q12. About how much financial debt do you have?

1. None
2. Less than $5,000
3. $5,000 - $10,000
4. $10,000 - $15,000
5. $15,000 - $20,000
6. More than $20,000

Q13. With whom do you currently live? **Select all that apply**.

1. I live alone
2. Parent(s) or Guardian(s)
3. Brother(s) and/or Sister(s)
4. Spouse or Domestic partner
5. My children
6. Other relatives
7. In a college dormitory
8. Other students in an apartment
9. Friends who are not students in an apartment
10. A shelter or other temporary housing
11. Other, please specify: ______________________

Q14. If you added together the income of **ALL** the people living and sharing expenses with you in your **primary household** in 2014 (*by primary household, we mean the place which you considered your main household*), **not** including scholarships or loans for school, would the **total** be….

- 1. Under $10,000
  2. $10,000 - $20,000
  3. $20,000 - $30,000
  4. $30,000 - $50,000
  5. $50,000 - $70,000
  6. $70,000 or more

Q15. Including yourself, how many people in your **PRIMARY HOUSEHOLD** are supported by this income? ____________ PEOPLE

Q16. Do you pay anything for the rent (or mortgage) to stay where you're now living?

1. Yes **→** Go to Q16a
2. No **→** Go to Q17

[If YES,] →

Q16a. Would you be able to stay where you are living now if what you had to pay was increased by $100.00 a month?

1. Yes, I could stay living there
2. No, I could not stay living there

**END SURVEY**
